# Influence of the Nucleo-Shuttling of the ATM Protein on the Response of Skin Fibroblasts from Marfan Syndrome to Ionizing Radiation

**DOI:** 10.3390/ijms252212313

**Published:** 2024-11-16

**Authors:** Dagmara Jakubowska, Joëlle Al-Choboq, Laurène Sonzogni, Michel Bourguignon, Dorota Slonina, Nicolas Foray

**Affiliations:** 1 Inserm, U1296 Unit, Radiation: Defense, Health and Environment, 28 rue Laennec, 69008 Lyon, France; dagmara.jakubowska@gliwice.nio.gov.pl (D.J.); joelle.al-choboq@inserm.fr (J.A.-C.); michel.bourguignon@inserm.fr (M.B.); 2 Maria Sklodowska-Curie National Research Institute of Oncology, Gliwice Branch, ul. Wybrzeże Armii Krajowej 15, 44-100 Gliwice, Poland; dorota.slonina@gliwice.nio.gov.pl; 3 Département de Biophysique et Médecine Nucléaire, Université Paris Saclay, Versailles St. Quentin-en-Yvelines, 78035 Versailles, France

**Keywords:** Marfan syndrome, FBN1, TGB-β, radiosensitivity, DNA double-strand breaks, ATM, ionizing radiation

## Abstract

Marfan syndrome (MFS) is an autosomal dominant connective-tissue disorder affecting multiple systems, such as skeletal, cardiovascular, and ocular systems. MFS is predominantly caused by mutations in the *FBN1* gene, which encodes the fibrillin-1 protein, crucial for connective-tissue integrity. *FBN1* mutations lead to defective fibrillin, resulting in structurally compromised connective tissues. Additionally, these mutations cause aberrant TGF-β expression, contributing to vascular issues and increased susceptibility to radiation-induced fibrosis. Studies about the potential radiosensitivity of MFS are rare and generally limited to case reports. Here, we aimed to investigate the radiation-induced ATM nucleo-shuttling (RIANS) model to explore the molecular and cellular radiation response in fibroblasts from MFS patients. The results showed that the MFS fibroblast cell lines tested are associated with moderate but significant radiosensitivity, high yield of micronuclei, and impaired recognition of DNA double-strand breaks (DSBs) caused by a diminished RIANS. The diminished RIANS is supported by the sequestration of ATM protein in the cytoplasm not only by mutated FBN1 protein but also by overexpressed TGF-β. This report is the first molecular and cellular characterization of the radiation response of MFS fibroblasts and highlights the importance of the FBN1-TGF-β complex after irradiation.

## 1. Introduction

Marfan syndrome (MFS) is an inherited autosomal dominant multi-systemic connective-tissue disorder, named after French pediatrician Antoine-Bernard Marfan who described a dolichostenomelia and an arachnodactylia in a 5-year-old girl in 1896 [1,2,3]. However, to date, the diagnosis of MFS has become enriched with articular hypermotility, aorta dilatation, valvular anomaly, lens luxation, and relies on identifying more specific physical traits, knowing that the MFS phenotype impacts different parts of the body such as the skeletal, cardiovascular, and ocular systems, exhibiting varying severity from selective skeletal manifestations to life-threatening aspects [1,2,4,5]. Severe cases involve multiple systems with rapidly progressive lesions, whereas mild MFS cases show isolated MFS characteristics [1]. Early diagnosis of MFS is important in preventing cardiovascular symptoms since 90% of the mortality cases are attributed to aortic dissection, rupture, or cardiac failure [1,4,5,6]. Other systems, like the lungs, skin, and central nervous system, might be involved in MFS phenotype but with less severe manifestations [1,7]. Estimated MFS prevalence ranges from 1/5000 to 1/10,000 individuals and remains consistent across diverse ethnicities and social classes [2,3].

At the molecular level, MFS is generally caused by dominantly inherited heterozygous mutations in the *FBN1* gene, a 230 kb gene, made up of 66 exons and located on chromosome 15q21.1 [1,7]. De novo *FBN1* mutations have also been described in sporadic cases, representing 20 to 30% of MFS cases. The *FBN1* gene encodes a ubiquitous connective-tissue microfibrillar protein named fibrillin-1 (FBN1) [3,7,8]. Fibrillins, akin to collagens, form visible fibers and are classified as “structural macromolecules”. FBN1 is an extracellular matrix glycoprotein and a structural component of microfibers widely distributed in elastic and nonelastic tissues [1,3]. *FBN1* mutations result in the production of abnormal fibrillin proteins whose incorporation into microfibrils along with normal fibrillin proteins results in structurally inferior connective tissue [3]. So far, identified mutations are distributed along the *FBN1* gene, and, to date, there is no evidence of a correlation between the mutation region and the clinical phenotype [7].

Another important feature of MFS is the role of the transforming growth factor beta (TGF-β). FBN1 spontaneously sequestrates TGF-β and inhibits its biological activity, thus playing an important role in controlling TGF-β-dependent pathways [9]. Conversely, mutations in *FBN1* are implicated in the upregulation of TGF-β [1,6]. Overexpressed TGF-β shows deleterious effects on vascular smooth muscle development and the integrity of the extracellular matrix [3], and an altered TGF-β signaling has been associated not only to MFS but also to other diseases like systemic sclerosis [10,11]. Besides, a very common risk factor for radiation treatments for cancer patients with MFS is radiation-induced fibrosis [6]. While the FBN-1/TGF-β may play a major role in systemic sclerosis, such a complex may be also a major feature of fibrillinopathies. Given the association between aberrant expression of TGF-β, that also serves as a pro-inflammatory cytokine, and radiation-induced fibrosis, patients with *FBN1* abnormalities, like those suffering from MFS and some systemic scleroderma, exhibit high susceptibility to radiation-induced (RI) fibrosis [6]. Furthermore, TGF-β actively participates in malignant transformation and progression and MFS was found to be associated with the development of certain solid malignancies, including thyroid cancer, osteosarcoma, and angiosarcoma [12]. 

The few studies available on the sensitivity to ionizing radiation of MFS are mainly based on case reports of patients exposed to anti-cancer radiotherapy [6,13,14]. The molecular basis of the radiation response of MFS fibroblasts has not been documented yet. Since 2014, our research group has built a mechanistic model of the human response to a large subset of ionizing radiation, based on the RI nucleo-shuttling of the ATM kinase, a major sensor of genotoxic stress (RIANS); RI oxidative stress leads to two important events: the induction of DNA double-strand breaks (DSB) in the nucleus and the monomerization of ATM dimers. Cytoplasmic ATM monomers diffuse thereafter from the cytoplasm to the nucleus, in which they phosphorylate the X variant of H2A histones at the site of the DSB by forming γH2AX foci, detectable and quantifiable by immunofluorescence. This recognition step is specific to DSB repaired by the non-homologous end-joining (NHEJ) pathway [15]. ATM monomers that phosphorylated H2AX reassemble at the DSB sites and form the trans-autophosphorylated nuclear ATM (pATM) foci, which is also quantifiable via immunofluorescence [15]. Diminished RIANS was shown to be associated with RI toxicity (radiosensitivity), RI cancers (radiosusceptibility), or RI-accelerated aging (radiodegeneration) [15,16]. The cause of diminished RIANS is generally the sequestration of ATM monomers in the cytoplasm by overexpressed ATM substrate proteins, called X-proteins, specific to each syndrome [15]. The radiobiological features of about twenty-five genetic syndromes have been already characterized in the frame of the RIANS model [15,17]. Here, we aimed to document the individual molecular and cellular responses of fibroblast cell lines derived from MFS patients to ionizing radiation by using RIANS biomarkers. To our knowledge, this study is the first radiobiological characterization of MFS cells.

## 2. Results

### 2.1. Spontaneous and RI Micronuclei in MFS Fibroblasts

**Figure 1:**
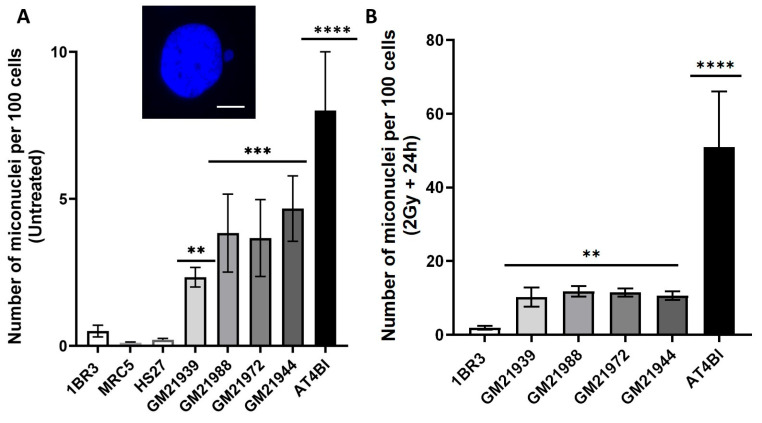
Micronuclei in MFS fibroblasts: Panel (**A**) shows the numbers of spontaneous micronuclei from the radioresistant 1BR3, MRC5 and HS27 controls and four MFS (GM21939, GM21988, GM21972, and GM21944) fibroblast cell lines. Panel (**B**) presents the number of micronuclei per 100 cells 24 h after 2 Gy X-rays from the radioresistant 1BR3, MRC5, and HS27 controls, hyper-radiosensitive *ATM*-mutated AT4BI, and four MFS (GM21939, GM21988, GM21972, and GM21944) fibroblast cell lines. Each plot represents the mean ± SEM of three replicates. The insert provides a representative example of a micronucleus observed via DAPI counterstaining. White bar represents 5 µm. Asteriks denote statistically significant differences from radioresistant control expressed as *p*-values (2, 3 and 4 asterisks correspond to *p* < 0.01, *p* < 0.001, and *p* < 0.0001, respectively).

Micronuclei (MNs) are small nuclei-like structures resulting from the encasement of chromosome fragments by the nuclear envelop during interphase [18]. These chromosome fragments usually arise from unrepaired DSB that spread through the cell cycle. Unrepaired chromosomal fragments are expelled from the nucleus during initial mitosis and are then extruded from the cell (exocytosis). The accumulation of MN has been widely used as a biomarker to assess genotoxic stress and genomic instability and was, notably correlated with the surviving fraction at 2 Gy (SF2), a reliable sensor of cellular radiosensitivity [19]. Here, MNs have been quantified using 4′,6-diamidino-2-phenylindole (DAPI) staining. The quantification of MNs was conducted before and 24 h after the exposure to 2 Gy X-rays. The 24 h data are usually taken as the maximal recovery time in terms of DNA repair [19]. The number of spontaneous MNs per 100 cells was significantly higher in the four MFS cells, in comparison with the radioresistant 1BR3 controls (*p* < 0.01) (Figure 1A). Concerning the RI MNs assessed 24 h after irradiation, the number of MNs per 100 cells was also significantly higher (*p* < 0.01) than that of radioresistant 1BR3 controls, and, again, lower than that observed in the hyper-radiosensitive *ATM*-mutated cells (*p* < 0.0001) (Figure 1B). All these findings suggest that MFS is associated with a significant amount of spontaneous and RI MNs, supporting both spontaneous and RI genomic instability. It is noteworthy that in all the conditions tested, the number of MN per cells or per binucleated cells is one or less. Indeed, in our hands, the events resulting in more than one MN per cell are due to higher doses or high concentrations of genotoxic drugs. 

In our previous works, we have documented the quantitative correlation between MN and SF2 for a large number of cells, including those from a tens of genetic diseases. From our database, MFS belongs to the syndromes associated with an intermediate cytogenetic (MN) and cellular (SF2) response to ionizing radiation (Figure S1A, Supplementary Materials). The predicted SF2 value deduced from our published correlation would range between 15 and 25%, supporting a moderate but significant cellular radiosensitivity associated with MFS. Furthermore, another correlation published elsewhere [19] has linked SF2 values with prevalence of syndromes. An estimated MFS prevalence ranging from 1/5000 to 1/10,000 would correspond to SF2 values ranging from 15 to 25%, supporting again that MFS is associated with a moderate but significant radiosensitivity (Figure S1B, Supplementary Materials)

### 2.2. Aberrant Numbers of γH2AX Foci Following Radiation Exposure in MFS Fibroblasts

**Figure 2:**
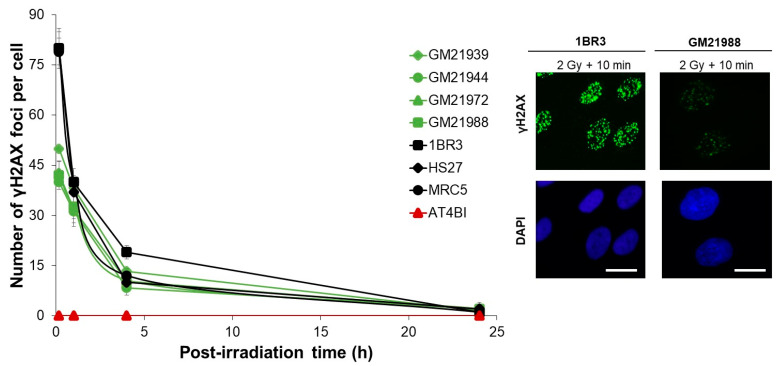
Kinetics of appearance/disappearance of γH2AX foci in MFS fibroblasts: The number of γH2AX foci is plotted against post-irradiation time. Data were obtained from the radioresistant 1BR3, MRC5, and HS27 controls; the hyper-radiosensitive *ATM*-mutated AT4BI; and four MFS (GM21939, GM21944, GM21972, and GM21988) fibroblast cell lines. Each plot represents the mean ± SEM of three independent experiments. The insert shows a representative illustration of DAPI-stained nuclei and γH2AX foci observed at 10 min post-irradiation (2 Gy X-rays) in the indicated cell lines. The white bar represents 10 µm.

As described in the Introduction and in the frame of the RIANS model, radiosensitivity may be caused by diminished RIANS. Therefore, in order to investigate the recognition and repair of RI DSB, we analyzed the kinetics of appearance and disappearance of γH2AX nuclear γH2AX foci. Early foci (i.e., assessed at 10 min and 1 h post-irradiation) reflect the recognition of DSB, whereas late γH2AX foci (i.e., assessed at 24 h post-irradiation) reflect the amount of unrepaired DSB managed by NHEJ [17]. In our cell culture conditions, the number of spontaneous γH2AX foci observed in the MFS cell lines was not significantly different from that of the radioresistant controls (*p* > 0.5). In the radioresistant controls, the number of γH2AX foci assessed 10 min post-irradiation averaged 79 ± 4 γH2AX foci per cell at 2 Gy, consistent with the previously published and consensual value of 37 ± 4 γH2AX foci per Gy per cell [17]. In agreement with published data, the number of γH2AX foci in the AT4BI cell line was found to be nil, in agreement with previous publications [17]. Conversely, the number of induced γH2AX foci assessed 10 min post-irradiation in the four MFS fibroblasts was found to be systematically lower than that observed in the radioresistant control (GM21939: 50 ± 1, GM21944: 40 ± 1, GM21972: 42.7 ± 3.7, and GM21988: 42.0 ± 4.1 γH2AX foci per cell; *p* < 0.001) (Figure 2), supporting an impaired DSB recognition in MFS cells. Conversely, the difference between the number of γH2AX foci observed 24 h post-irradiation in MFS fibroblasts and the controls was not found significant (*p* > 0.1). All these results suggest that MFS fibroblasts are characterized by a diminished RIANS, causing a poor recognition of RI DSB by NHEJ. However, the amount of DSB recognized and repaired by NHEJ are similar to those of the radioresistant controls. Hence, the subset of DSB that are not recognized by H2AX phosphorylation may be responsible for the molecular and cellular radiosensitivity of MFS cells. 

### 2.3. Abnormal Number of pATM Foci After Irradiation in MFS Fibroblasts 

As highlighted earlier, the number of induced H2AX foci observed 10 min after irradiation in MFS fibroblasts consistently appeared lower compared to the radioresistant control cells. These findings, as previously noted, do not imply a lesser induction of DSB in MFS cells but less DSB recognized by the NHEJ pathway [15]. In the framework of the RIANS model, cytoplasmic dimeric forms of ATM monomerize in response to radiation. ATM monomers diffuse into the nucleus and bind to the DSB sites. At the end of the repair process, ATM monomers trans-autophosphorylate and re-dimerize at the DSB sites, forming pATM nuclear foci quantifiable by immunofluorescence [15]. We therefore measured, using the same immunofluorescence conditions, the formation of nuclear pATM foci, in order to verify whether the low number of γH2AX is due to a diminished RIANS. 

**Figure 3:**
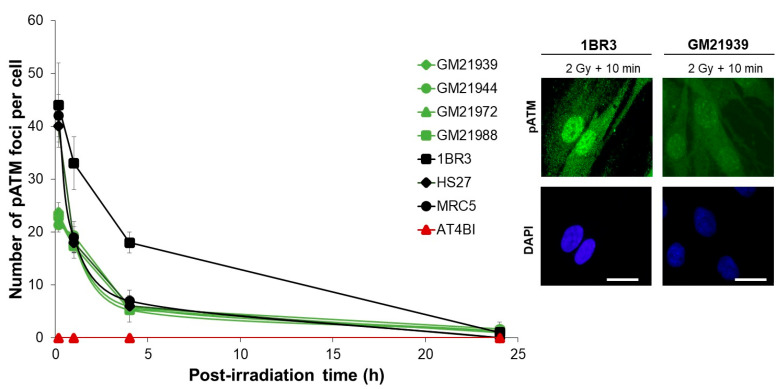
Kinetics of appearance/disappearance of pATM foci in MFS fibroblasts: The number of pATM foci was plotted against post-irradiation time. Data were obtained from the radioresistant 1BR3, MRC5, and HS27 controls; the hyper-radiosensitive *ATM*-mutated AT4BI; and four MFS (GM21939, GM21944, GM21972, and GM21988) fibroblast cell lines. Each plot represents the mean ± SEM of three replicates. The insert shows a representative illustration of DAPI-stained nuclei and pATM foci observed at 10 min post-irradiation (2 Gy X-rays) in the indicated cell lines. The white bar represents 10 µm.

According to our historical data, exposure to 2 Gy X-rays typically results in approximately 40 pATM foci per cell at 10 min post-irradiation in radioresistant controls. This number gradually diminishes with repair time, reaching insignificance at 24 h post-irradiation. In the present study, the radioresistant 1BR3 control cell line showed 44 ± 8 and 1 ± 2 pATM foci per cell at 10 min and 24 h post-irradiation, respectively (Figure 3). The other radioresistant cell lines elicited similar data (*p* > 0.5). In agreement with published data, the number of pATM foci in AT4BI cell line was found to be nil. The number of pATM foci observed in MFS fibroblasts 10 min post-irradiation significantly differed from that in the control fibroblasts (GM21939: 23.7 ± 1.85, GM21988: 23.0 ± 1.52, GM21972: 23.7 ± 1.85, and GM21944: 21.3 ± 1.33 pATM foci per cell compared to 44 ± 8; *p* < 0.02) (Figure 3). However, similar to γH2AX data, no significant difference was observed at 24 h post-irradiation, compared to radioresistant controls (*p* > 0.5). 

### 2.4. Abnormal Number of MRE11 Foci After Irradiation in MFS Fibroblasts

**Figure 4:**
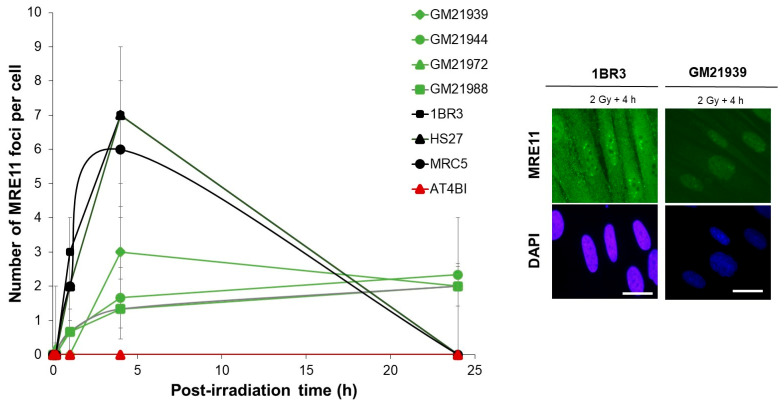
Kinetics of appearance/disappearance of MRE11 foci in MFS fibroblasts. The number of MRE11 foci was plotted against post-irradiation time. Data were obtained from the radioresistant 1BR3, MRC5, and HS27controls; the hyper-radiosensitive *ATM*-mutated AT4BI; and the four MFS (GM21939, GM21944, GM21971, and GM21988) fibroblast cell lines. Each plot represents the mean ± SEM of three independent experiments. The insert shows a representative illustration of DAPI-stained nuclei and MRE11 foci observed at 4 h post-irradiation in the indicated cell lines. The white bar represents 10 µm.

The MRE11 protein, a constituent of the RAD50-MRE11-NBS1 complex, is typically phosphorylated and inhibited by ATM and forms nuclear foci following genotoxic stress. We therefore examined MRE11 foci kinetics to reinforce our data described above. Previous examinations revealed an evident impairment in the formation of the MRE11 foci post-irradiation in *ATM*-mutated cells, which exhibited a distinct pattern of MRE11 foci [17]. In the radioresistant controls, MRE11 foci were observed between 2 and 8 h post-irradiation, peaking at 4 h (7 ± 2 MRE11 foci per cell). The kinetics of MRE11 foci in the four MFS fibroblasts displayed a different profile compared to the radioresistant controls, with a maximum number of MRE11 foci observed at 4 h post-irradiation (Figure 4). These numbers, however, never matched the peak value seen in the radioresistant controls (GM21939: 3.0 ± 0.3, GM21988: 1.3 ± 0.88, GM21972: 1.3 ± 0.88, and GM21944: 1.7 ± 0.33 compared to 1BR3: 7 ± 2, *p* < 0.05). At 24 h post-irradiation, the number of MRE11 foci was different from zero for all four tested MFS cell lines, potentially suggesting a persistence of DNA breaks and therefore pro-inflammatory events. Overall, these observations indicate an abnormal formation of MRE11 foci in MFS fibroblasts after exposure to ionizing radiation, suggesting an exacerbated MRE11 nuclease activity. 

### 2.5. Subcellular Localization and Expression of the FBN1 Protein in MFS Fibroblasts

**Figure 5:**
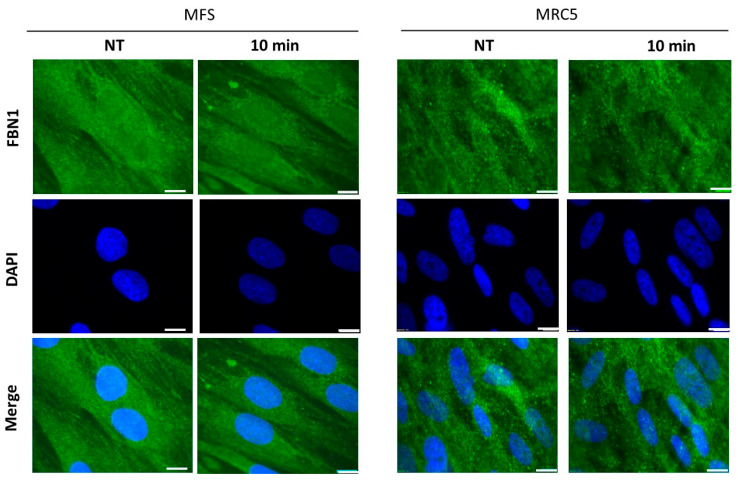
Subcellular localization of FBN1 proteins in MFS and control fibroblasts. Anti-*FBN1* immunofluorescence was applied to the GM21939 and the MRC5 fibroblasts, either before or after irradiation (2 Gy X-rays). The white bars represent 10 µm.

In the frame of the RIANS model, a diminished RIANS is associated with overexpressed cytoplasmic proteins, substrates of ATM (i.e., holding SQ or TQ domains), called X-proteins. X-proteins are generally the gene product mutated responsible for the syndrome. Here, the potential X-protein for MFS is the FBN1 protein. Interestingly, the FBN1 protein holds 3 SQ and 2 TQ domains. In order to examine the existence of potential cytoplasmic ATM-FBN1 complexes in MFS fibroblasts and the involvement of FBN1 protein in the cellular response to irradiation, its subcellular localization was examined through an immunofluorescence analysis. In control cells, the FBN1 signal appeared to be weak and essentially cytoplasmic. In all the tested MFS fibroblasts, a stronger cytoplasmic and nuclear localization of FBN1 protein was observed before and after 2 Gy X-rays (Figure 5). These findings confirm the hypothesis of a cytoplasmic expression of FBN1 protein in MFS fibroblasts.

**Figure 6:**
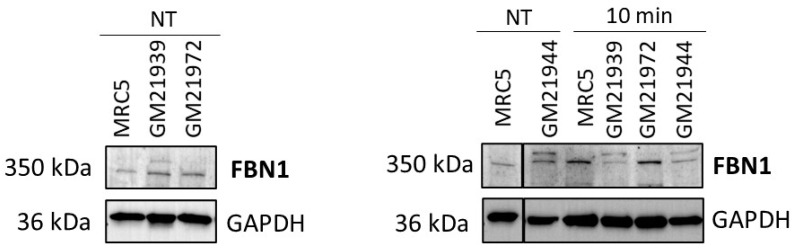
Expression of the FBN1 protein in MFS fibroblasts by using immunoblotting. Anti-*FBN1* immunoblots with cytoplasmic protein extracts of the indicated non-irradiated (NT) and irradiated (10 min after irradiation) MFS and control fibroblasts tested in this study.2.6. FBN1 protein interacts with ATM abundantly in MFS fibroblasts.

As a second step, we examined the FBN1 protein expression by immunoblots in cytoplasmic cell extracts before and after irradiation. As presented in Figure 6, the three tested MFS cell lines (GM21939, GM21972, and GM21944) showed more FBN1 protein expression in comparison with the control cell line before irradiation. Interestingly, to the notable exception of the GM21972 cell line, an extra band was observed in the MFS fibroblasts. This extra band has not been described yet; it can be a non-specific band or a band corresponding to a phosphorylated form of FBN1. However, further investigations are needed to document one of these hypotheses.

**Figure 7:**
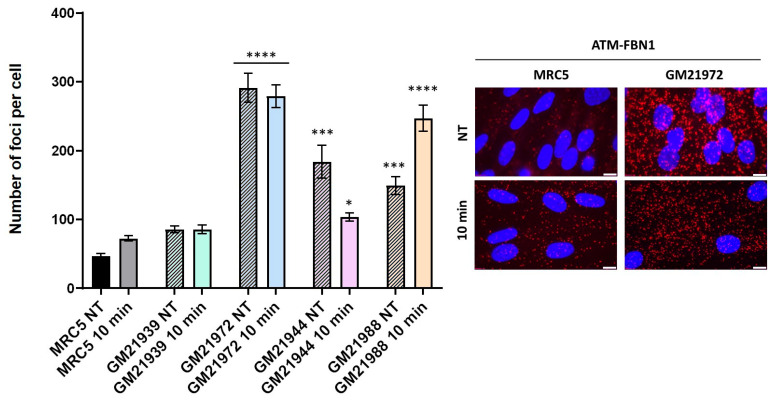
Interaction between ATM and FBN1 protein through proximity ligation assay (PLA), applied to the indicated cell lines. Representative PLA images from GM21972 fibroblasts reveal cytoplasmic and some nuclear PLA signals. The quantification of red foci, indicative of cytoplasmic ATM-FBN1 protein complexes, was determined per 100 cells. Each data point represents the mean ± SEM from at least two independent experiments. Nuclei were counterstained with DAPI (blue). Asterisks denote statistically significant differences from radioresistant control, expressed as *p*-values (1, 3 and 4 asterisks correspond to *p* < 0.05, *p* < 0.001, and *p* < 0.0001, respectively). The white bars represent 10 µm.

RIANS was found diminished in MFS fibroblasts. We hypothesized therefore that this delay is the consequence of the formation of ATM-FBN1 complexes. Hence, we investigated the presence and localization of these ATM-FBN1 complexes in MFS fibroblasts by using proximity ligation assay (PLA). The resulting images exhibited numerous marked red foci within the cytoplasm of MFS cell lines and confirmed the existence of cytoplasmic ATM-FBN1 complexes, with varying abundance across different MFS cell lines (Figure 7). The number of these complexes was significantly higher in MFS cells than in radioresistant control cells, whether before or after irradiation. Overall, our findings strongly suggest the existence of ATM-FBN1 complexes particularly abundant in MFS cells, supporting again the delay of the RIANS (Figure 7).

### 2.6. TGF-β Protein Interacts with ATM Abundantly in MFS Fibroblasts

**Figure 8:**
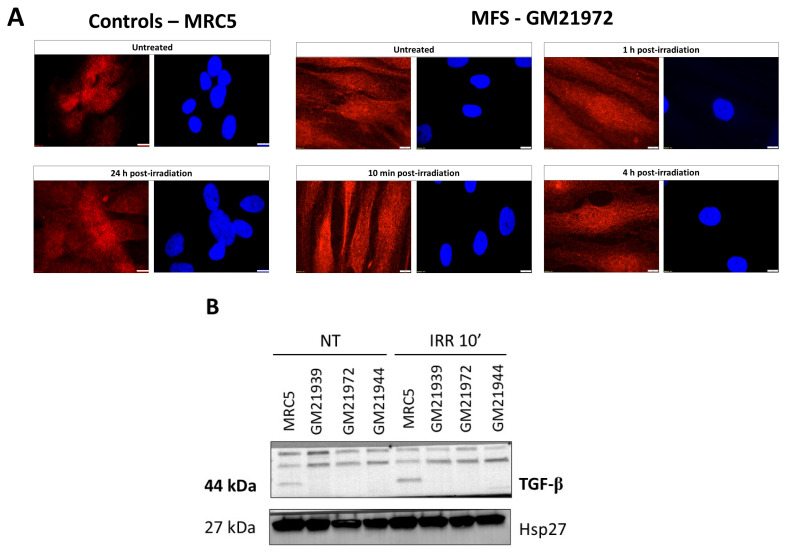

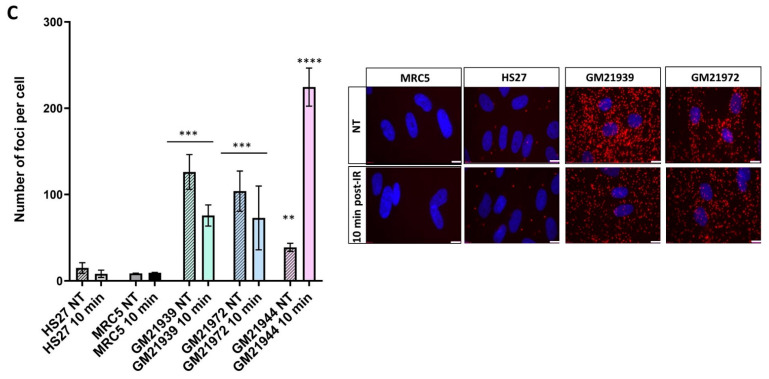
Interaction between TGF-β and ATM in MFS fibroblasts. (**A**). TGF-β subcellular localization in MFS GM21972 and MRCR5 controls fibroblasts by immunofluorescence using anti-TGF-β antibody whether before or after 2 Gy X-rays. The white bar represents 5 µm. (**B**). Expression of TGF-β in cytoplasmic protein extracts by immunoblotting in the indicated controls and MFS fibroblasts, before and 24 h after irradiation (2 Gy X-rays). For technical reasons, the GM21988 data were not available for this endpoint. (**C**). Interaction between ATM and TGF-β protein through proximity ligation assay (PLA), applied to the indicated cell lines. Representative PLA images from GM21939 and GM21972 fibroblasts reveal mostly cytoplasmic PLA signals. The quantification of red foci, suggesting cytoplasmic ATM-TGF-β protein complexes, was determined per 100 cells. Each data point represents the mean ± SEM from at least two independent experiments. Nuclei were counterstained with DAPI (blue). Asterisks denote statistically significant differences from radioresistant controls, expressed as *p*-values (2, 3, and 4 asterisks correspond to *p* < 0.01, *p* < 0.001, and *p* < 0.0001, respectively). The white bar represents 10 µm.

As evoked in the Introduction, the wild-type FBN1 protein spontaneously sequestrates TGF-β and inhibits its biological activity. Conversely, mutations in *FBN1* are implicated in the upregulation of TGF-β [1,6]. Interestingly, TGF-β holds one SQ and one TQ domain, which makes highly probable the phosphorylation of TGF-β by ATM. Additionally, it was shown that ATM and TGF-β are involved in the same DNA damage signaling pathway [20]. We hypothesized therefore that the diminished RIANS caused by the formation of ATM-FBN1 complexes may be also amplified by the formation of ATM-TGF-β complexes in cytoplasm. To verify such a hypothesis, we first applied immunofluorescence to document the subcellular localization of TGF-β in MFS fibroblasts. Figure 8A shows that TGF-β is abundantly expressed in MFS fibroblasts both in nucleus and in cytoplasm, whether before or after irradiation. Interestingly, after irradiation, some MFS fibroblasts may show a perinuclear form of TGF-β (Figure 8A). The abundance of TGF-β in MFS fibroblasts was also confirmed with immunoblots, whatever the conditions of irradiation. Interestingly, unlike the radioresistant control, the MFS cell extracts showed two bands on immunoblots. The origin of these two bands is unknown (Figure 8B).

By applying PLA to MFS fibroblasts, the resulting images confirmed the existence of cytoplasmic ATM-TGF-β complexes, with varying abundance across different MFS cell lines. Interestingly, the number of these complexes appeared higher before irradiation. Overall, our findings strongly suggest the existence of ATM-TGF-β complexes particularly abundant in MFS cells, supporting again the delay of the RIANS.

### 2.7. Treatment with Statins and Bisphosphonates Protects MFS Fibroblasts from Radiation

**Figure 9:**
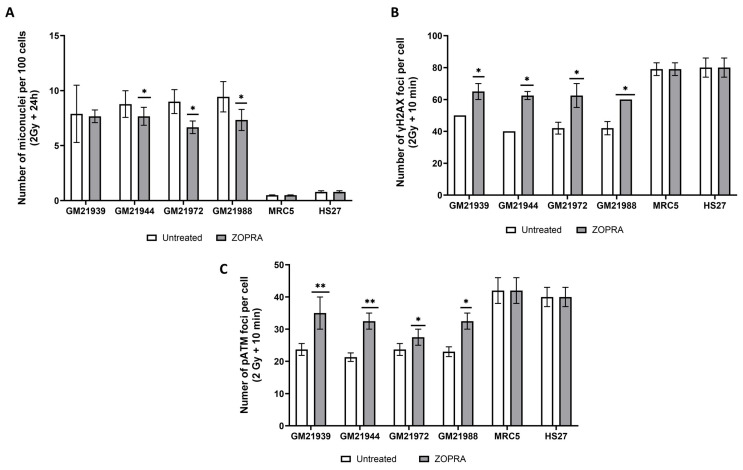
Effect of ZOPRA treatment on MFS fibroblasts in response to radiation. Number of micronuclei per 100 cells assessed after 2 Gy X-rays followed by 24 h post-irradiation (**A**), number of ɣH2AX foci per cell assessed after 2 Gy X-rays followed by 10 min post-irradiation (**B**), and number of pATM foci per cell assessed after 2 Gy X-rays followed by 10 min post-irradiation (**C**) in radioresistant MRC5 and HS27 controls and in GM21939, GM21944, GM21972, and GM21988 fibroblasts, with or without ZOPRA treatment. Each data point represents the mean ± SEM of at least two independent experiments. Asterisks represent the statistically significant differences from non-treated MFS fibroblasts, expressed as *p*-values (1 and 2 asterisks correspond to *p* < 0.05 and *p* < 0.01, respectively).

In our previous reports, the combination of pravastatin and zoledronate (ZOPRA) has been shown to stimulate the RIANS and decrease radiosensitivity in various genetic diseases like Huntington’s disease, tuberous sclerosis complex syndrome, neurofibromatosis type 1 syndrome, and Xeroderma Pigmentosum D [21,22]. We therefore examined the effect of the ZOPRA treatment on MFS cells. It is very well documented that the ZOPRA treatment showed no significant impact on MN yields or on the kinetics of γH2AX, pATM, and MRE11 foci in radioresistant control cells [17]. By contrast, the ZOPRA treatment significantly decreased the number of micronuclei in two MFS fibroblast cell lines (GM21944 and GM21972) 24 h after irradiation (Figure 9A). Regarding the γH2AX data, there was also a significant increase in the number of γH2AX foci assessed 10 min post-irradiation in the four MFS fibroblast cell lines (Figure 9B). Concerning the pATM data, the ZOPRA treatment led to an increase in pATM foci assessed 10 min post-irradiation in all MFS fibroblast cell lines tested (Figure 9C). Altogether, these findings suggest that the ZOPRA treatment may increase RIANS, thus increasing the recognition of DSB and protect MFS fibroblasts from irradiation. Further investigations are needed to consolidate these findings.

## 3. Discussion

### 3.1. The MFS Fibroblasts Show Significant Molecular and Cellular Radiosensitivity

Our data suggest a significant radiosensitivity of the MFS fibroblast cell lines tested, associated with a high yield of spontaneous and residual micronuclei and abnormal γH2AX, pATM and MRE11 foci kinetics after irradiation with a significant diminished RIANS. The ZOPRA data also suggest that the delay of the RIANS may be reversible. The diminished RIANS in MFS fibroblast cell lines was likely due to the formation of ATM-FBN1 and ATM-TGF-β complexes in the cytoplasm. Since MFS patients may present some risk of cancer like thyroid cancer, osteosarcoma, and angiosarcoma [12,23], some of these patients may be exposed to anti-cancer radiotherapy and/or chemotherapy and may show RI fibroses already documented in the literature [13,14]. Hence, our findings raise the question of the risks of adverse tissue reactions post-radiotherapy or post-chemotherapy and even potential risk of RI cancers that would be specific to MFS patients: the radiodiagnosis and low doses of ionizing radiation may reveal the impairment of RIANS of MFS patients and increase the risks of RI cancer. The radiobiological characterization of the MFS and the role of the mutated FBN1 protein, as well as the overexpressed TGF-β in the DNA damage repair and signaling, are therefore of both medical and scientific interest. However, we are aware that even though our data converge to the same conclusion, this report is based on only four MFS fibroblast cell lines. It is, however, noteworthy that the RIANS model and its biomarkers have permitted quantitative inter-correlations of radiobiological parameters relevant on more 200 fibroblast cell lines, eliciting a large spectrum of radiosensitivity [19].

To the notable exception of the present study, the radiosensitivity associated with MFS was mainly based on some case reports with clinical observations of RI fibroses. A recent report pointed out the cumulative radiation dose and lifetime cancer risk in MFS patients who underwent computed tomography angiography of the aorta [24]. To our knowledge, this study is one of the first documentations of the molecular and cellular responses of MFS cells to ionizing radiation. Our investigations were based on the RIANS model validated on several radiosensitive genetic diseases [17]. As already specified, the RIANS model consists of several steps: (1) in response to RI oxidative stress, cytoplasmic ATM dimers dissociate in ATM monomers in proportion of stress; (2) these ATM monomers diffuse into the nucleus; (3) in the nucleus, ATM monomers phosphorylate the X variant of the H2A histone around the DSB sites, the first step of DSB recognition [25]. Early foci (10 min after irradiation) represent the recognition of DSB: the faster this step, the more radioresistant the cells (group I of radiosensitivity). A delay of the RIANS—likely caused by the formation of protein complexes with ATM—will cause a phenotype of significant but moderate radiosensitivity (called group II). In some, but rare, cases, the DSB repair pathway can be impaired, either at the recognition step (e.g., when *ATM* is mutated) or the repair step (e.g., when *LIG4* is mutated). These cases represent the most hyper-radiosensitive ones (group III of radiosensitivity). The group III syndromes are generally caused by the suppression of a biological function of the mutated protein caused by homozygous mutations, while group II syndromes are caused by heterozygous mutations [15]. From these definitions, MFS is caused by heterozygous mutations of the *FBN1* gene. All our findings and notably the quantitative values obtained strongly suggest that MFS can be considered as a group II syndrome. 

### 3.2. The FBN1 and the TGF-β Proteins Interact with ATM and May Influence the RIANS

**Figure 10:**
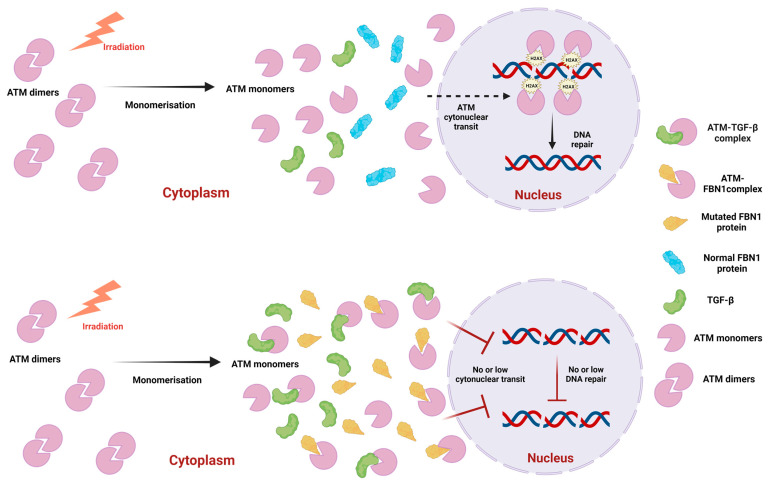
Mechanistic model for the RIANS in MFS fibroblasts. Upper panel: In radioresistant cells, in response to radiation, cytoplasmic ATM dimers monomerize, and ATM monomers diffuse into the nucleus, where they phosphorylate H2AX histones at DSB sites, thus triggering DSB recognition. Components of DNA repair pathway will therefore be activated and DSB repaired. Lower panel: In the MFS fibroblast, the cytoplasmic mutated FBN1 protein (in orange), as well as the overexpressed TGF-β protein (in green), both serve as ATM substrates and form complexes with ATM monomers in the cytoplasm. These complexes cause a delay in the repair pathway of radiation-induced DSB, causing the radiosensitivity of MFS fibroblasts. (Created with BioRender.com (accessed on 15 August 2024)).

Radiation-induced fibroses observed in patients treated with radiotherapy for cancer has so far been linked to the overexpression of TGF-β. But how can we explain the link between the radiosensitivity observed in MFS fibroblasts with two proteins, TGF-β and FBN1, that do not play a direct role in DNA damage repair pathway? As demonstrated in several genetic syndromes associated with radiosensitivity and belonging to the group II [17], the ATM monomers are sequestrated in the cytoplasm by over-expressed ATM substrates, called X-proteins. An X-protein must reach two requirements, at least: (1) to be over-expressed in the cytoplasm; (2) to hold putative SQ/TQ ATM phosphorylation domains allowing it to associate with ATM. Interestingly, both FBN1 and TGF-β proteins obey this double conditions. The fact that a mutation of *FBN1* can dissociate the FBN1-TGF-β complexes may explain how the number of potential X-proteins can be doubled in MFS cells. Recall again that overexpressed TGF-β shows deleterious effects on vascular smooth muscle development and the integrity of the extracellular matrix [3]. A molecular model based on the RIANS theory can therefore be developed to describe the specific response to ionizing radiation of MFS cells, as Figure 10 shows it. 

## 4. Materials and Methods

### 4.1. Cell Culture 

All the experiments were performed with human untransformed cutaneous fibroblasts that were routinely cultured at 37 °C in 5% CO_2_ humid conditions as monolayers with Dulbecco’s modified Eagle’s minimum medium (DMEM) (Gibco-Invitrogen-France, Cergy-Pontoise, France), supplemented with 20% fetal calf serum, penicillin, and streptomycin (Sigma-Aldrich, Saint-Quentin-Fallavier, France). All the experiments were performed with cells in the plateau phase of growth to avoid any cell cycle effect [17]. The four untransformed MFS fibroblasts, GM21939, GM21988, GM21972, and GM21944, were purchased from the Coriell Institute for Medical Research (Candem, NJ, USA) (Table 1). Three radioresistant controls, 1BR3, MRC5, and Hs27, originating from apparently healthy donors and one hyper-radiosensitive *ATM*-mutated AT4BI originating from an ataxia telangiectasia patient were used in this study. These cell lines belong to the abundantly documented COPERNIC collection, approved by a regional ethical committee described elsewhere [25]. This collection obeys the French regulations about anonymous sampling and informed consent. Cell authentication and culture quality control were insured by commercial repositories. Concerning the cell lines isolated from the laboratory (COPERNIC collection), the respect for the good practices of the laboratory (GPL) was included in the declaration of the cell line collection to the French Ministry of Research.

### 4.2. Treatment with Zoledronate and Pravastatin (ZOPRA)

The combined administration of zoledronate and pravastatin, referred to as ZOPRA treatment and detailed elsewhere [17,22].

### 4.3. Irradiation

All the irradiations were performed on a 6 MeV X-ray clinical irradiator (SL 15 Philips) at the Anti-Cancer Centre Léon Bérard (Lyon, France) at a dose of 2 Gy with a dose rate of 6 Gy·min^−1^. Dosimetry was certified by the Radiophysics Department of Centre Léon Bérard.

### 4.4. Immunofluorescence

The immunofluorescence- and foci-scoring techniques have been detailed elsewhere [17]. In summary, the anti-γ*H2AX^ser139^* antibody (#05-636-1, clone JBW301; Merck, Millipore, Darmstadt, Germany) was used at a concentration of 1:800. The anti-*pATM^ser1981^* (ab78, Abcam, Cambridge, UK), anti-*MRE11* (#56211; Abcys, Paris, France), and anti-TGF-β (A15103, Abclonal, Düsseldorf, Germany) antibodies were applied at a 1:100 concentration. 

### 4.5. Micronuclei Assay

The use of 4′,6-diamidino-2-phenylindole, dihydrochloride (DAPI) counterstaining enabled the quantification of micronuclei at a magnification of ×100 [19]. The micronuclei data were obtained from the analysis of 100 nuclei per experiment and per biomarker; three independent experiments were performed.

### 4.6. Cell Extracts and Immunoblotting 

Cell extract and immunoblots techniques have been documented elsewhere [17]. Briefly, total extracts were obtained using the following lysis buffer: 50 mM Tris, pH 8, 150 mM NaCl, 2 mM EDTA, pH 8, 10% glycerol, 0.2% Nonidet NP40, H2O. Cytoplasmic extracts were obtained after applying the following lysis buffer: 10 mM Hepes pH 7.9, 1.5 mM MgCl_2_, 10 mM KCL, 2 mM ethylene diaminetetraacetic acid (EDTA) pH 8, 0.5 mM dithiothreitol (DTT), 0.2% Nonidet NP40, H2O. Both buffers were supplemented with protease and phosphatase inhibitors (#78442, Thermo Fisher, Waltham, MA, USA). Protein concentrations were measured with a Bio-Rad Bradford assay (Bio-Rad Laboratories, Hercules, CA, USA), and aliquots for extracts were stored at −20 °C. Proteins were subjected to SDS–polyacrylamide gel electrophoresis (SDS-PAGE) and blotted onto polyvinylidene fluoride (PVDF) membranes (Immobilon-P, Millipore). Membranes were blocked in Tris-buffered saline (TBS) solution containing 0.05% Tween 20 and 5% (*w*/*v*) non-fat, dried, skimmed milk powder and incubated with primary antibodies for 3 h and horseradish-peroxidase-conjugated secondary antibodies (Jackson ImmunoResearch, West Grove, PA, USA) for 1 h. Antibody binding was determined using Clarity Max ECL substrate (#1705061, Bio-Rad Laboratories) and/or a SuperSignal West Femto Maximum Sensitivity Substrate (#34095, Thermo Scientific, Waltham, MA, USA). Analysis of Western blot bands was performed using ImageLab software version 6.1 (Bio-Rad Laboratories, Hercules, CA, USA). 

### 4.7. In Situ Proximity Ligation Assay (PLA)

The Proximity Ligation Assay (PLA) enables the in situ detection of endogenous protein interactions through immunofluorescence microscopy [26,27]. The protocol applied in this paper has been described elsewhere (Ref Usher). The antibodies used were mouse monoclonal anti-*ATM* antibody (#2C1 (1A1); Abcam, Cambridge, UK), rabbit polyclonal anti-FBN1 (PA5-99225, Invitrogen, Walthman, MA, USA) and rabbit polyclonal anti-TGF-β (A15103, Abclonal, Düsseldorf, Germany). 

### 4.8. Statistical Analysis

Statistical analyses were performed using PRISM software version 9.5.1(GraphPad Software, San Diego, CA, USA) or Kaleidagraph version 4.5.4 (Synergy Software, Reading, PA, USA).

## 5. Conclusions

Our findings showed that the MFS fibroblast cell lines tested are associated with moderate but significant radiosensitivity, high yield of micronuclei, and impaired recognition of DNA double-strand breaks (DSB) caused by a diminished RIANS. The diminished RIANS is supported by the sequestration of ATM protein in the cytoplasm not only by mutated FBN1 protein but also by overexpressed TGF-β. We emphasize that this study was conducted on only four MFS fibroblastic cell lines. Hence, further experiments are needed to better quantify the risk caused by DNA breaking agents (radiotherapy, radiodiagnosis, chemotherapy) on a larger cohort of healthy and MFS cell lines. 

## Figures and Tables

**Table 1:** A summary of MFS cell lines characteristics as presented on the Coriell Institute website.

DMD Cell Line	Phenotypic Data	Identified Mutation
GM21939	Bilateral lens dislocation. Adult height of 186 cm with long arms and legs, narrow highly arched palate, dental crowding, chest asymmetry and pectus deformity, arthritis: thin fragile skin, myopia, glaucoma.	DEL EX42-43 of the FBN1 gene
GM21988	Dolichostenomelia, severe pectus carinatum, high narrow palate, dental crowding, contractures, myopia, bilateral ectopia lentis, ascending aortic aneurysm, aortic dissection.	6185insA of the FBN1 gene
GM21972	Scoliosis, high narrow palate, dental crowding, hypermobile small joints, hypermobile large joints, arachnodactyly, myopia, bilateral ectopia lentis, ascending aortic aneurysm at age 42 years, severe mitral valve prolapse; mitral regurgitation; incisional hernia; striae.	2399delC of the FBN1 gene
GM21944	Pectus carinatum, high narrow palate, hypermobile small joints, hypermobile large joints, positive wrist sign, positive thumb sign; arachnodactyly; myopia; ectopia lentis; ascending aortic aneurysm; aortic root replacement at age 7 years; mitral valve prolapse; mitral regurgitation; pneumothorax	CYS1326ARG

## Data Availability

All the data can be provided on reasonable request.

## Supplementary Materials

The supporting information can be downloaded at: https://www.mdpi.com/article/10.3390/ijms252212313/s1.

## References

[1] Du Q., Zhang D., Zhuang Y., Xia Q., Wen T., Jia H. (2021). The Molecular Genetics of Marfan Syndrome. Int. J. Med. Sci..

[2] Gonzales E.A. (2009). Marfan syndrome. J. Am. Acad. Nurse Pract..

[3] Kumar A., Agarwal S. (2014). Marfan syndrome: An eyesight of syndrome. Meta Gene.

[4] Milewicz D.M., Braverman A.C., De Backer J., Morris S.A., Boileau C., Maumenee I.H., Jondeau G., Evangelista A., Pyeritz R.E. (2021). Marfan syndrome. Nat. Rev. Dis. Primers.

[5] Zeigler S.M., Sloan B., Jones J.A. (2021). Pathophysiology and Pathogenesis of Marfan Syndrome. Adv. Exp. Med. Biol..

[6] Blakaj D.M., Zhang H.G., Blakaj A., Mourad W.F., Clarke B., Spierer M., Kalnicki S., Guha C. (2013). Clinical and molecular exploration of the impact of radiation therapy on Marfan syndrome patients. Pract. Radiat. Oncol..

[7] Comeglio P., Johnson P., Arno G., Brice G., Evans A., Aragon-Martin J., da Silva F.P., Kiotsekoglou A., Child A. (2007). The importance of mutation detection in Marfan syndrome and Marfan-related disorders: Report of 193 FBN1 mutations. Hum. Mutat..

[8] Sakai L.Y., Keene D.R., Renard M., De Backer J. (2016). FBN1: The disease-causing gene for Marfan syndrome and other genetic disorders. Gene.

[9] Kaartinen V., Warburton D. (2003). Fibrillin controls TGF-β activation. Nat. Genet..

[10] Chaudhry S.S., Cain S.A., Morgan A., Dallas S.L., Shuttleworth C.A., Kielty C.M. (2007). Fibrillin-1 regulates the bioavailability of TGFbeta1. J. Cell Biol..

[11] Ayers N.B., Sun C.M., Chen S.Y. (2018). Transforming growth factor-β signaling in systemic sclerosis. J. Biomed. Res..

[12] Hsu C.W., Wang J.C., Liao W.I., Chien W.C., Chung C.H., Tsao C.H., Wu Y.F., Liao M.T., Tsai S.H. (2017). Association between malignancies and Marfan syndrome: A population-based, nested case-control study in Taiwan. BMJ Open.

[13] Suarez E.M., Knackstedt R.J., Jenrette J.M. (2014). Significant fibrosis after radiation therapy in a patient with Marfan syndrome. Radiat. Oncol. J..

[14] Finlay M., Laperriere N., Bristow R.G. (2005). Radiotherapy and Marfan syndrome: A report of two cases. Clin. Oncol. (R. Coll. Radiol.).

[15] Berthel E., Foray N., Ferlazzo M.L. (2019). The Nucleoshuttling of the ATM Protein: A Unified Model to Describe the Individual Response to High- and Low-Dose of Radiation?. Cancers.

[16] Foray N., Bourguignon M., Hamada N. (2016). Individual response to ionizing radiation. Mutat. Res. Rev. Mutat. Res..

[17] Al-Choboq J., Ferlazzo M.L., Sonzogni L., Granzotto A., El-Nachef L., Maalouf M., Berthel E., Foray N. (2022). Usher Syndrome Belongs to the Genetic Diseases Associated with Radiosensitivity: Influence of the ATM Protein Kinase. Int. J. Mol. Sci..

[18] Krupina K., Goginashvili A., Cleveland D.W. (2021). Causes and consequences of micronuclei. Curr. Opin. Cell Biol..

[19] Le Reun E., Bodgi L., Granzotto A., Sonzogni L., Ferlazzo M.L., Al-Choboq J., El-Nachef L., Restier-Verlet J., Berthel E., Devic C. (2022). Quantitative Correlations between Radiosensitivity Biomarkers Show That the ATM Protein Kinase Is Strongly Involved in the Radiotoxicities Observed after Radiotherapy. Int. J. Mol. Sci..

[20] Li Y., Liu Y., Chiang Y.J., Huang F., Li Y., Li X., Ning Y., Zhang W., Deng H., Chen Y.G. (2019). DNA Damage Activates TGF-β Signaling via ATM-c-Cbl-Mediated Stabilization of the Type II Receptor TβRII. Cell Rep..

[21] Ferlazzo M., Berthel E., Granzotto A., Devic C., Sonzogni L., Bachelet J.T., Pereira S., Bourguignon M., Sarasin A., Mezzina M. (2020). Some mutations in the xeroderma pigmentosum D gene may lead to moderate but significant radiosensitivity associated with a delayed radiation-induced ATM nuclear localization. Int. J. Radiat. Biol..

[22] Varela I., Pereira S., Ugalde A.P., Navarro C.L., Suarez M.F., Cau P., Cadinanos J., Osorio F.G., Foray N., Cobo J. (2008). Combined treatment with statins and aminobisphosphonates extends longevity in a mouse model of human premature aging. Nat. Med..

[23] Maya-González C., Delgado-Vega A.M., Taylan F., Lagerstedt Robinson K., Hansson L., Pal N., Fagman H., Puls F., Wessman S., Stenman J. (2024). Occurrence of cancer in Marfan syndrome: Report of two patients with neuroblastoma and review of the literature. Am. J. Med. Genet. A.

[24] Chaosuwannakit N., Aupongkaroon P., Makarawate P. (2022). Determine Cumulative Radiation Dose and Lifetime Cancer Risk in Marfan Syndrome Patients Who Underwent Computed Tomography Angiography of the Aorta in Northeast Thailand: A 5-Year Retrospective Cohort Study. Tomography.

[25] Granzotto A., Benadjaoud M.A., Vogin G., Devic C., Ferlazzo M.L., Bodgi L., Pereira S., Sonzogni L., Forcheron F., Viau M. (2016). Influence of Nucleoshuttling of the ATM Protein in the Healthy Tissues Response to Radiation Therapy: Toward a Molecular Classification of Human Radiosensitivity. Int. J. Radiat. Oncol. Biol. Phys..

[26] Ristic M., Brockly F., Piechaczyk M., Bossis G. (2016). Detection of Protein-Protein Interactions and Posttranslational Modifications Using the Proximity Ligation Assay: Application to the Study of the SUMO Pathway. Methods Mol. Biol..

[27] Fredriksson S., Gullberg M., Jarvius J., Olsson C., Pietras K., Gústafsdóttir S.M., Ostman A., Landegren U. (2002). Protein detection using proximity-dependent DNA ligation assays. Nat. Biotechnol..

