# Supplementary material for: Influence of the Nucleo-Shuttling of the ATM Protein on the Response of Skin Fibroblasts from Marfan Syndrome to Ionizing Radiation
Source: Int J Mol Sci. 2024 Nov 16;25(22):12313. doi: 10.3390/ijms252212313 (PMC11594578; doi:10.3390/ijms252212313)
Supplement: Supplementary file 1 [file ijms-25-12313-s001.zip › ijms-3212787-supplementary.pdf]

Influence of the Nucleo-Shuttling of the ATM Protein on the Response of Skin Fibroblasts from Marfan Syndrome to Ionizing Radiation

Dagmara Jakubowska [1,2,†], Joëlle Al-Choboq [1,†], Laurène Sonzogni [1], Michel Bourguignon [1,3], Dorota Slonina [2] and Nicolas Foray [1,*]

[1] Inserm, U1296 Unit, Radiation: Defense, Health and Environment, 28 rue Laennec, 69008 Lyon, France; dagmara.jakubowska@gliwice.nio.gov.pl (D.J.); joelle.al-choboq@inserm.fr (J.A.-C.); michel.bourguignon@inserm.fr (M.B.)
[2] Maria Sklodowska-Curie National Research Institute of Oncology, Gliwice Branch, ul. Wybrzeże Armii Krajowej 15, 44-100 Gliwice, Poland; dorota.slonina@gliwice.nio.gov.pl
[3] Département de Biophysique et Médecine Nucléaire, Université Paris Saclay, Versailles St. Quentin-en-Yvelines, 78035 Versailles, France
* Correspondence: nicolas.foray@inserm.fr
† These authors contributed equally to this work.

Supplementary Data

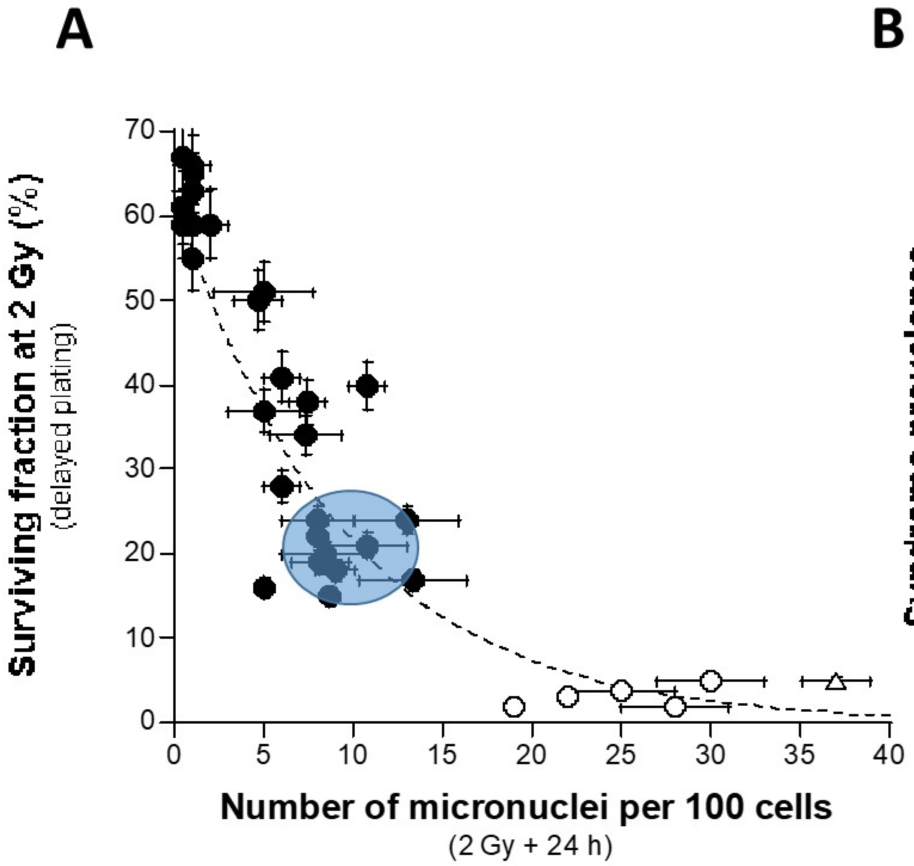

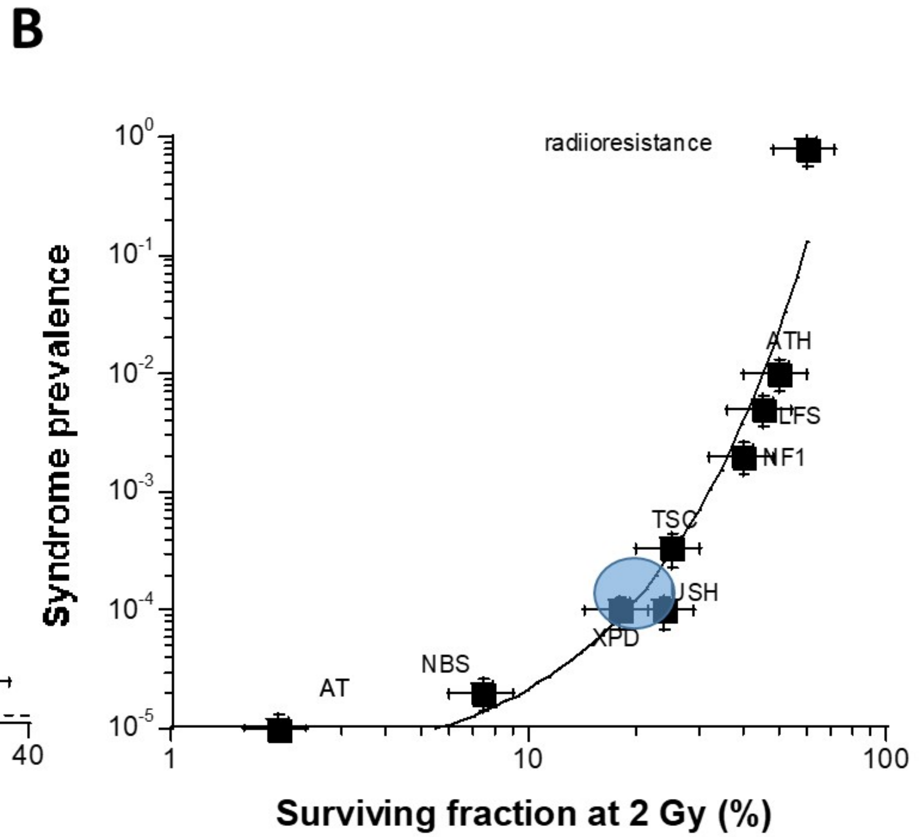

Figure S1: Correlation between surviving fraction at 2 Gy (SF2) with number of RI micronuclei and syndrome prevalence. A. SF2 data from the (26) reference were plotted against their corresponding values of the number of micronuclei (2 Gy + 24 h). Each plot represents the mean of triplicates ± standard error of the mean (SEM). The blue confidence zone has been built from the micronuclei data from MFS fibroblasts with the hypothesis that MSF data obey thegeneral correlation (dashed line). B. SF2 data from the (26)

reference were plotted against their corresponding prevalence. Each plot represents the mean of triplicates ± standard error of the mean (SEM). The blue confidence zone has been built from the MFS prevalence (literature data) and with the hypothesis that MFS data obey the general correlation (solid line). The SF2 values deduce dfrom the two panels A and B are in agreement.
